# Health Care Access, Socioeconomic Status, and Acute Kidney Injury Outcomes: A Prospective National Study

**DOI:** 10.1016/j.xkme.2024.100867

**Published:** 2024-07-10

**Authors:** Ali AlSahow, Omar Alkandari, Anas AlYousef, Bassam AlHelal, Heba AlRajab, Ahmed AlQallaf, Yousif Bahbahani, Monther AlSharekh, Abdulrahman AlKandari, Gamal Nessim, Bassem Mashal, Ahmad Mazroue, Alaa Abdelmoteleb, Mohamed Saad, Ali Abdelzaher, Emad Abdallah, Mohamed Abdellatif, Ziad ElHusseini, Ahmed Abdelrady

**Affiliations:** 1 Division of Nephrology, Jahra Hospital, Al Jahra, Kuwait; 2 Division of Pediatric Nephrology, Mubarak Hospital, Jabriya, Kuwait; 3 Division of Nephrology, Amiri Hospital, Kuwait City, Kuwait; 4 Division of Nephrology, Adan Hospital, Hadiya, Kuwait; 5 Division of Nephrology, Farwaniya Hospital, Kuwait City, Kuwait; 6 Division of Nephrology, Jaber Hospital, Kuwait City, Kuwait; 7 Division of Nephrology, Mubarak Hospital, Jabriya, Kuwait; 8 Division of Nephrology, Chest Diseases Hospital, Kuwait City, Kuwait

**Keywords:** Acute kidney injury, dialysis, Kuwait, mortality, socioeconomic disparity

## Abstract

**Rationale & Objectives:**

Acute kidney injury (AKI) incidence and outcome in Kuwait are unknown. Moreover, non-Kuwaitis, who represent 66% of the population, have lower income, and their access to public health services is restricted compared with Kuwaitis who have free full access.

**Study Design:**

Observational prospective multicenter cohort study.

**Setting & Participants:**

Adult inpatients with AKI in 7 public hospitals from January 1 to December 31, 2021.

**Exposure:**

AKI identified using Kidney Disease: Improving Global Outcomes serum creatinine-based criteria.

**Outcomes:**

For hospitalized patients with AKI, the outcomes included 30-day outcomes of mortality, need for dialysis, kidney recovery rates, and differences in outcomes between Kuwaitis and non-Kuwaitis.

**Analytical Approach:**

A backward stepwise multiple logistic regression analysis was performed to assess possible independent risk factors for the outcomes.

**Results:**

We recruited 3,744 patients (mean age: 63 years; mean baseline estimated glomerular filtration rate [eGFR]: 66.7 mL/min; non-Kuwaitis: 42.3%), representing 3.2% of hospitalizations and 19.5% of intensive care unit (ICU) admissions. Non-Kuwaitis were significantly younger (57.6 vs 66.9 years), with higher baseline eGFR (73.1 vs. 62 mL/min), more frequent community acquired AKI (53.8% vs 46.7%), and AKI in summer (34.7% vs 26.9%). Dialysis was provided to 33.5% of patients, with a higher need for non-Kuwaitis (35.5% vs 32.1%). At 30 days, 34.4% of patients died, representing 24.8% of hospital mortality and 59.8% of ICU mortality. No differences in mortality or kidney recovery were noted between Kuwaitis and non-Kuwaitis. Low eGFR did not affect the mortality rate.

**Limitations:**

Observational nature and short follow-up period of 30 days only.

**Conclusions:**

AKI was associated with high dialysis need and mortality. Non-Kuwaitis accounted for less cases despite representing 66% of the population because they were younger with higher baseline eGFR and fewer comorbid conditions. Non-Kuwaitis had higher rates of community acquired AKI and AKI in summer and a higher need for dialysis but had similar mortality and complete kidney recovery rates.

## Plain-Language Summary

Incidences of acute kidney injury (AKI), its management, and its outcomes are unknown in Kuwait. In addition, Kuwait has a large population of ethnically diverse expatriates who have lower income and do not enjoy the same level of access to public hospital services. We recruited hospitalized adults who have a diagnosis of AKI in several public hospitals in Kuwait. We analyzed characteristics, management, and outcomes data for more than 3,700 patients and found that AKI affects 3.2% of hospitalized patients. AKI leads to high dialysis utilization rates and causes high mortality rates. Although more Kuwaitis were affected by AKI, the mortality rates for Kuwaitis and non-Kuwaitis were similar. Non-Kuwaitis were younger with better baseline kidney function and fewer chronic diseases than Kuwaitis.

Acute kidney injury (AKI), which is characterized by an abrupt increase in serum creatinine levels leads to significant morbidity and mortality.^1^^,^^2^ However, little is known about AKI incidence and outcomes in Kuwait. In addition, Kuwait has a large ethnically diverse non-Kuwaiti (expatriate) community, representing 66% of the total population of 4,385,717 in 2021.^3^ Expatriates are noncitizens with temporary residency permit that can be extended indefinitely. The ministry of health (MoH) provides the bulk of health care for the population of Kuwait. Kuwaitis have full free access to all MoH outpatient and inpatient services with laboratory and radiology services as well as prescriptions provided free of charge. Expatriates must pay for an annual MoH insurance card to access MoH services. Other applicable fees paid upfront include fees for visit to the emergency department or outpatient clinic (which covers the cost of basic blood tests and the prescription of certain medications). Admission to MoH hospitals for expatriates, whether urgent or elective, and inpatient services, including surgical interventions, vascular access procedures, preoperative evaluation (anesthesia, cardiology, etc.), and acute dialysis (intermittent or continuous), are provided free of charge. However, they need to pay for the hospitalization stay (fee is per night and goes up for a private room in general ward or a bed in an intensive care unit [ICU]). In addition, radiology services and kidney biopsy are also provided at a fee, albeit subsidized. Such fees are kept on hold for hospitalized patients until after discharge. A charity group helps cover some of the cost, and they are expected to repay the remaining amount. If they cannot, then they may receive an exemption or extension under special circumstances. However, they often cannot pay, and MoH takes no further action. Most expatriates have a lower income than Kuwaitis, cannot afford private health care because of its prohibitive cost, and are forced into substandard housing conditions and consumption of cheap unhealthy food. The majority are also working more physically demanding jobs at longer hours. Moreover, many expatriates elect to visit public primary care clinics instead of hospitals, where they pay much less per visit than for hospital visit, mainly to get a refill of part of their permissible medications and part of the permissible blood tests (primary care clinic cannot replace hospital services), without proper follow-up, which may affect outcome.^4^ Poor access to health care increases the risk for kidney disease,^5^ and low socioeconomic status is associated with higher AKI incidence, more severe AKI, and higher mortality.^6^ We aimed to study AKI in Kuwait; to evaluate the differences in characteristics, management, and outcomes of AKI between Kuwaitis and non-Kuwaitis; and to assess whether the lower income and restricted access to public health care services have an effect on outcomes.

## Methods

This was a prospective study that recruited nephrology consultations for AKI in adult inpatients above the age of 18 years with native kidneys and an estimated baseline glomerular filtration rate (eGFR) above 10 mL/min admitted to any of 7 MoH hospitals in Kuwait from January 1 to December 31, 2021. Kidney transplant recipients, dialysis patients, and chronic kidney disease (CKD) patients not receiving dialysis but with eGFR < 10 mL/min (or even higher if they have pre-emptive hemodialysis access) were excluded. We also excluded pre-AKI (recovery in <24 hours) and patients who died within 24 hours of consultation. Patients’ demographics, clinical profiles, and management data were collected. Patients were followed up for 30 days (or less if recovery or death occurs earlier), even after discharge to record outcomes. Kidney Disease: Improving Global Outcome (KDIGO) definitions and staging of AKI^2^ and CKD^7^ for patients with pre-existing CKD were used. The cohort was divided into 2 subgroups defined by access to public health care: Kuwaiti patients and non-Kuwaiti patients (expatriates).

For statistical analysis, mean ± standard deviations (SDs) were used for continuous variables, and numbers/percentages were used for categorical variables for descriptive statistics of the study population. Here, *t* tests, Mann Whitney Wilcoxon test, or Kruskal–Wallis tests were used for contingency table analysis, as appropriate, whereas Pearson’s χ^2^ - χ^2^ tests or Fisher exact tests were used to compare categorical variables. Univariable logistic regression analysis was used to evaluate the possible risk factors associated with 3 outcomes: mortality, need for dialysis, and lack of kidney recovery. We included the following variables in the model for the multivariable regression analysis: sex, age > 65 years, and comorbid conditions (diabetes, hypertension and baseline eGFR). Subsequently, a backward stepwise multiple logistic regression analysis (*P* value exit criterion = 0.05) was performed to assess the possible independent risk factors for the previously mentioned 3 outcomes. The following factors were included in the model (nationality, community acquired AKI, baseline hemoglobin level, intravenous fluid use, diuretics use, vasopressor use, steroid use, mechanical ventilation, surgical intervention and dialysis need. A *P* value of < 0.05 was determined to be statistically significant. STATA statistical software version 17 (Stata Corp, LLC) was used for statistical analysis. Study was approved by the MoH–Kuwait University Joint Committee on Medical and Scientific Research on (2019/1242 issued on February 13, 2020). The Strengthening the Reporting of Observational Studies in Epidemiology (STROBE) guidelines for cohort studies were followed.^8^

## Results

Table 1 describes AKI patients basic characteristics. The total number of recruited patients was 3,744 (mean age: 63 years; males: 59.2%; mean baseline eGFR: 66.7 mL/min; community acquired cases: 49.7%). Non-Kuwaitis accounted for 42.3% of all patients. They were significantly younger (57.6 vs 66.9 years) with higher baseline eGFR (73.1 vs 62 ml/min), fewer comorbid conditions, higher rates of community acquired AKI (53.8% vs 46.7%), and higher rates of AKI occurring in the hot summer months (34.7% of cases in June, July, and August) than other seasons, whereas Kuwaitis showed no seasonal variation (26.9% of cases in the summer). Volume depletion and renin angiotensin aldosterone system inhibitors were significantly more important precipitating factors for AKI in non-Kuwaitis than in Kuwaitis. Pre-existing CKD (regardless of baseline eGFR) was reported in 47.1% of patients (64.4% were Kuwaitis), with diabetes mellitus (DM) as the most common cause. COVID-19 was reported in 24.2% of cases, and AKI occurred in only 3.9% of the total hospitalized population with COVID-19.

**Table 1:** Basic Characteristics of Patients With Acute Kidney Injury

	Total cohort N = 3,744	Kuwaiti N = 2,159 (57.7%)	Non-Kuwaiti N = 1,585 (42.3%)	*P* value
Age, y, mean ± SD	63.0 ± 16.2	66.9 ± 15.8	57.6 ± 15.2	<0.001
Males, n (%)	2,218 (59.2%)	1,079 (50.0%)	1,139 (71.9%)	<0.001
Baseline eGFR level, mL/min, mean ± SD	66.7 ± 31.3	62.0 ± 28.0	73.1 ± 34.2	<0.001
Baseline hemoglobin level , g/L, mean ± SD	105.1 ± 23.5	102.8 ± 21.6	108.2 ± 25.5	<0.001
Community acquired AKI, n (%)	1,861 (49.7%)	1,009 (46.7%)	852 (53.8%)	<0.001
Comorbid conditions^a^				
Age > 65 y, n (%)	1,827 (48.8%)	1,320 (61.1%)	507 (32.0%)	<0.001
Diabetes, n (%)	2,611 (69.7%)	1,694 (78.5%)	917 (57.9%)	<0.001
Hypertension, n (%)	2,720 (72.7%)	1,720 (79.7%)	1,000 (63.1%)	<0.001
Cardiovascular disease^b^, n (%)	1,778 (47.5%)	1,175 (54.4%)	603 (38.0%)	<0.001
Obesity (BMI > 30), n (%)	310 (8.3%)	179 (8.3%)	131 (8.3%)	0.97
Gastrointestinal and liver diseases, n (%)	349 (9.3%)	195 (9.0%)	154 (9.7%)	0.5
Urological disorders, n (%)	266 (7.1%)	128 (5.9%)	138 (8.7%)	0.001
History of AKI within the past 12 months, n (%)	209 (5.6%)	115 (5.3%)	94 (5.9%)	0.43
No comorbid conditions/unknown past medical history, n (%)	419 (11.2%)	127 (5.9%)	292 (18.4%)	<0.001
Pre-existing CKD^a^^,^^c^^,^^e^	1,763 (47.1%)	1,135 (52.6%)	628 (39.6%)	<0.001
Diabetic kidney disease, n (%)	987 (26.4%)	714 (33.1%)	273 (17.2%)	<0.001
Hypertension and vasculopathy, n (%)	171 (4.6%)	134 (6.2%)	37 (2.3%)	<0.001
Glomerulopathies, n (%)	258 (6.9%)	155 (7.2%)	103 (6.5%)	0.4
Urological disorders, n (%)	61 (1.6%)	28 (1.3%)	33 (2.1%)	0.06
Others or unknown, n (%)	421 (11.2%)	217 (10.1%)	204 (12.9%)	0.007
Cause of AKI^a^^,^^d^				
Ischemic or toxic ATN, n (%)	3,325 (88.8%)	1,978 (91.6%)	1,347 (85.0%)	<0.001
Drug-related injury, n (%)	441 (11.8%)	245 (11.4%)	196 (12.4%)	0.34
Glomerular disorders, n (%)	464 (12.4%)	341 (15.8%)	123 (7.8%)	<0.001
COVID-19 related, n (%)	907 (24.2%)	538 (24.9%)	369 (23.3%)	0.25
Others, n (%)	527 (14.1%)	251 (11.6%)	276 (17.4%)	<0.001
Precipitating factors^a^				
Volume depletion, n (%)	2,515 (67.1%)	1,404 (65.0%)	1,111 (70.1%)	0.001
RAASi, n (%)	318 (8.5%)	162 (7.5%)	156 (9.8%)	0.01
Drug toxicity, n (%)	729 (19.5%)	422 (19.5%)	307 (19.4%)	0.9
Sepsis, n (%)	2,371 (63.3%)	1,429 (66.1%)	942 (59.5%)	<0.001
Other, n (%)	197 (5.3%)	69 (3.1%)	128 (8.1%)	<0.001

Values are given as mean ± SD for continuous variables and as number (percentage) for categorical variables.

Abbreviations: eGFR, estimated glomerular filtration rate; AKI, acute kidney injury; BMI, body mass index; CKD, chronic kidney disease; ATN, acute tubular necrosis; RAASi, renin angiotensin aldosterone inhibitors (angiotensin converting enzyme inhibitor [ACEi] and angiotensin receptor blocker [ARB]).

a More than one answer per patient was possible, which explains why the total may be >100%.

b Cardiovascular disease: Coronary artery disease, heart failure, peripheral arterial disease.

c Pre-existing diagnosis of CKD, regardless of baseline eGFR, before AKI.

d Kidney biopsy was performed in only 3% of all AKI cases.

e Kidney biopsy was performed in 9.4% of patients with pre-exciting CKD.

Table 2 details AKI management. Intravenous (IV) fluids were provided to 80.4% of patients. In addition, IV loop diuretics were prescribed to only 44.9%, and IV vasopressors were prescribed to 42.3%. There was no difference in the use of IV fluids between Kuwaitis and non-Kuwaitis; however, IV loop diuretics were used less frequently in non-Kuwaitis. ICU AKI cases accounted for 42.9% of the cohort, and 40.5% of AKI patients needed mechanical ventilation. No differences in either ICU admission or ventilation requirement were noted between the 2 groups. However, dialysis need was significantly higher for non-Kuwaitis than Kuwaitis (35.5% vs 32.1%). Volume overload was a much more frequent indication for Kuwaitis. Dialysis vascular access of choice was the right internal jugular in 53.8%, with no difference between the 2 groups. Continuous kidney replacement therapy (CKRT) was the initial modality of choice in 90.9% of cases; however, 16.6% of those patients were on CKRT because of lack of water treatment unit in the inpatient wards in some hospitals preventing intermittent hemodialysis (HD) utilization and not because of clinical indication. Only 8.5% of CKRT patients were later switched to intermittent HD, and 1% of intermittent HD patients were switched to CKRT. Dialysis was indicated but not performed (was considered futile or was refused by the family) in only 3.7% of cases.

**Table 2:** Nondialytic and Dialytic Management of Acute Kidney Injury

	Total cohort N = 3,744	Kuwaiti N = 2,159 (57.7%)	Non-Kuwaiti N = 1,585 (42.3%)	*P* value
IV fluids^a^				
None, *n* (%)	735 (19.6%)	452 (20.9%)	283 (17.8%)	0.02
Normal saline, *n* (%)	2,269 (60.6%)	1,326 (61.4%)	943 (59.5%)	0.2
Bicarbonate, Ringer’s lactate, Plasma-Lyte, *n* (%)	954 (25.5%)	458 (21.2%)	496 (31.3%)	<0.001
Blood/blood products/albumin, *n* (%)	545 (14.6%)	315 (14.6%)	230 (14.5%)	0.95
Any IV fluid or combination, *n* (%)	3,009 (80.4%)	1,707 (79.1%)	1,302 (82.2%)	0.02
IV diuretics^a^				
None, *n* (%)	2,048 (54.7%)	1,131 (52.4%)	917 (57.9%)	0.001
Loop diuretics, *n* (%)	1,681 (44.9%)	1,019 (47.2%)	662 (41.8%)	0.001
Thiazide diuretics, *n* (%)	93 (2.5%)	49 (2.3%)	44 (2.8%)	0.3
Potassium-sparing diuretics, *n* (%)	27 (0.72%)	17 (0.8%)	10 (0.6%)	0.6
Any IV diuretic or combination, *n* (%)	1,696 (45.3%)	1,028 (47.6%)	668 (42.2%)	<.001
IV vasopressors^a^				
None, *n* (%)	2,159 (57.7%)	1,214 (56.2%)	945 (59.6%)	0.04
Noradrenaline, *n* (%)	1,414 (37.8%)	850 (39.4%)	564 (35.6%)	0.02
Vasopressin, *n* (%)	531 (14.2%)	320 (14.8%)	211 (13.3%)	0.2
Dopamine, *n* (%)	285 (7.6%)	145 (6.7%)	140 (8.8%)	0.02
Dobutamine, *n* (%)	72 (1.9%)	43 (2.0%)	29 (1.8%)	0.7
Any IV vasopressor or combination, *n* (%)	1,585 (42.3%)	945 (43.8%)	640 (40.4%)	0.04
Steroid use, n (%)	1,038 (27.7%)	608 (28.2%)	430 (27.1%)	0.5
Antibiotic use, n (%)	2,930 (78.3%)	1,691 (87.3%)	1239 (78.2%)	0.9
Urgent urology/surgery intervention, *n* (%)	536 (14.3%)	266 (12.3%)	270 (17.0%)	<0.001
Admission to ICU, *n* (%)	1,605 (42.9%)	925 (42.8%)	680 (42.9%)	0.9
Mechanical ventilation, *n* (%)	1,516 (40.5%)	890 (41.2%)	626 (39.5%)	0.3
ECMO, *n* (%)	121 (3.2%)	63 (2.9%)	58 (3.7%)	0.2
Dialysis indication^a^	1,255 (33.5%)	693 (32.1%)	562 (35.5%)	0.03
Volume overload, *n* (%)	873 (69.6%)	537 (77.5%)	336 (59.8%)	<0.001
Hyperkalemia and acid–base disorders, *n* (%)	1,029 (82.0%)	568 (82.0%)	461 (82.0%)	0.97
Uremia, *n* (%)	199 (15.9%)	105 (15.2%)	94 (16.7%)	0.5
Initial dialysis modality	1,255	693	562	
Intermittent hemodialysis, *n* (%)	113 (9.1%)	41 (5.9%)	72 (12.8%)	<0.001
CKRT, *n* (%)	1,142 (91.0%)	652 (94.1%)	490 (88.4%)	0.01
Dialysis vascular access				
Right internal jugular, *n* (%)	675 (53.8%)	370 (53.4%)	305 (54.3%)	0.75
Left internal jugular, *n* (%)	191 (15.2%)	117 (16.9%)	74 (13.2%)	0.07
Femoral (right or left), *n* (%)	385 (30.6%)	203 (29.2%)	182 (32.4%)	0.2
Subclavian (right or left) , *n* (%)	4 (0.32%)	3 (0.4%)	1 (0.18%)	0.4

Abbreviations: IV, intravenous; ICU, intensive care unit; ECMO, extracorporeal membrane oxygenation; CKRT, continuous kidney replacement therapy.

a More than one answer per patient was possible, which explains why the total may be >100%.

Table 3 details kidney and patient outcomes. At 30 days, 34.4% of the total cohort had died (58.9% were Kuwaitis), with no difference between the 2 groups. ICU patients accounted for 71.6% of deaths in the cohort. Almost 80% of patients who did not receive dialysis survived, whereas only 37.5% of those who required dialysis survived. Also, at 30 days, 57.1% of dialyzed patients died while still receiving dialysis, and only 17.8% came off dialysis. After excluding deceased patients, complete kidney recovery (ie, a return to baseline eGFR) occurred in 54.3% of patients, and only 9% of those who received dialysis made a complete recovery. Lack of recovery (ie, unchanged serum creatinine value from nadir or failure to wean off dialysis for patients who received dialysis) was reported in 17.4% of patients who remained alive. There were no differences between the 2 groups in dialysis outcomes or in rates of recovery of kidney function. The mean final eGFR at 30 days for patients who were alive and not treated with dialysis was 54 mL/min and was significantly higher in non-Kuwaitis than Kuwaitis (58.3 vs. 50.7 mL/min).

**Table 3:** Patient and Kidney Outcomes at 30 Days After Consultation

	Total cohort N = 3,744	Kuwaiti N = 2,159 (57.7%)	Non-Kuwaiti N = 1,585 (42.3%)	*P* value
Cause of death^a^	1,289 (34.4%)	759 (35.2%)	530 (33.4%)	0.27
Infection/sepsis, n (%)	993 (77.0%)	618 (81.4%)	375 (70.8%)	<0.001
Respiratory, n (%)	355 (27.5%)	246 (32.4%)	109 (20.6%)	<0.001
Cardiovascular, n (%)	215 (16.7%)	119 (15.7%)	96 (18.1%)	0.25
Other, n (%)	131 (10.2%)	68 (9.0%)	63 (11.9%)	0.09
Dialysis status at 30 days for dialyzed patients	1,255 (100%)	693 (32.1%)	562 (35.5%)	0.03
Still receiving dialysis, n (%)	223 (17.8%)	127 (18.3%)	96 (17.1%)	0.6
Further dialysis not needed, n (%)	316 (25.2%)	170 (24.5%)	146 (26.0%)	0.6
Died while receiving dialysis, n (%)	716 (57.1%)	396 (57.1%)	320 (56.9%)	0.9
Kidney function at 30 days^b^	2,455 (100%)	1,400 (57.0%)	1,055 (43.0%)	-
No recovery, n (%)	426 (17.4%)	259 (18.5%)	167 (15.8%)	0.08
Partial recovery, n (%)	695 (28.3%)	392 (28.0%)	303 (28.7%)	0.7
Complete recovery, n (%)	1,333 (54.3%)	748 (53.4%)	585 (55.5%)	0.3
eGFR at 30 days, mL/min, mean ± SD^c^	54.0 ± 29.4	50.7 ± 27.8	58.3 ± 30.9	<0.001

Values are given as mean ± SD for continuous variables and as number (percentage) for categorical variables.

Abbreviation: eGFR, estimated glomerular filtration rate.

a More than one answer per patient was possible, which explains why the total may be >100% and > total deaths of 1,289.

b N = 2,454; after excluding deceased patients.

c N = 2,233; after excluding deceased patients and patients still receiving dialysis at 30 days.

Table 4 details the AKI contribution to admissions and mortality in both groups in participating hospitals during 2021. AKI was a diagnosis in 3.2% of all hospital admissions and in 19.5% of all ICU admissions. Non-Kuwaitis accounted for 47% of all hospital admissions and 39.6% of all ICU admissions. AKI contributed to 24.8% of total hospital mortality, 17.3% of total mortality outside the ICU, and 59.8% of total ICU mortality in participating hospitals during the study period.

**Table 4:** Incidences During the Study Period from January 1 to December 31, 2021 in All Participating Hospitals Combined

Variable	Total	Kuwaitis	Non-Kuwaitis
Total hospital admissions, *n*	117,467	53%	47%
AKI proportion of total hospital admissions, *n* (%)	3,744 (3.2%)	57.7%	42.3%
Total ICU admissions, *n*	8,220	60.4%	39.6%
AKI proportion of total ICU admissions, *n* (%)	1,605 (19.5%)	60.4%	39.6%
Total mortality of all hospitalized patients, *n* (%)	5,216 (4.4%)	56%	44%
AKI-related mortality of total hospital mortality, *n* (%)	1,289 (24.8%)	58.9%	41.1%
K AKI-related mortality of total K hospital mortality, %	-	26.1%	-
NK AKI-related mortality of total NK hospital mortality, %	-	-	23%
Total mortality of all ICU patients, *n* (%)	1,549 (18.8%)	60%	40%
ICU AKI-related mortality of total ICU mortality, *n* (%)	923 (59.8%)	57.6%	42.4%
K ICU AKI-related mortality of total K ICU mortality, %	-	57%	-
NK ICU AKI-related mortality of total NK ICU mortality, %	-	-	63%

Abbreviations: AKI, acute kidney injury; ICU, intensive care unit; K, Kuwaiti; NK, non-Kuwaiti.

Significant factors associated with increased mortality after adjustment (Table 5) included age >65 years; hospital-acquired AKI; mechanical ventilation; the use of fluids, diuretics, vasopressors, and steroids; and the need for dialysis. Significant factors associated with increased need for dialysis after adjustment (Table 6) included eGFR < 60; community acquired AKI; mechanical ventilation; and the use diuretics, vasopressors and steroids. DM was associated with an increased need for dialysis in patients with eGFR ≥ 60 mL/min. Significant factors associated with lack of recovery of kidney function after adjustment (Table 7) included eGFR < 60; hypertension (HTN); community acquired AKI; use of steroids; need for surgical intervention; and the need for dialysis. Lower hemoglobin levels were only slightly associated with less kidney recovery. Given that 24.2% of patients were infected with coronavirus disease (COVID)-19, a subanalysis of the 3 outcomes using the same variables but adjusted for COVID-19 was performed and showed no effect of COVID-19 on mortality (data not shown).

**Table 5:** Risk Factors Associated With Higher Mortality

Variable	Total cohort N = 3,744	Deceased N = 1,289	Unadjusted^b^ OR (95% CI)	Adjusted^b^ OR (95% CI)
Male sex, n (%)	2,218 (59.2%)	753 (58.4%)	0.95 (0.82-1.08)	1.05 (0.87-1.26)
Comorbid conditions^a^				
Age > 65 y, n (%)	1,827 (48.8%)	676 (52.4%)	1.25 (1.09-1.43)	1. 76 (1.45-2.15)
eGFR < 60 mL/min, n (%)	1,563 (41.8%)	456 (35.4%)	0.6 (0.52-0.69)	0.73 (0.6-0.89)
DM, n (%)	2,611 (69.7%)	877 (68.0%)	0.88 (0.76-1.02)	0.93 (0.74-1.18)
HTN, n (%)	2,720 (72.7%)	905 (70.2%)	0.83 (0.71-0.96)	0.99 (0.78-1.25)
Nationality Kuwaiti, n (%)	2,159 (57.7)	759 (58.9)	1.08 (0.94-1.23)	
Baseline Hgb level, g/L, mean ± SD	105.1 ± 23.5	102.3 ± 22.3	0.992 (0.989-0.995)	
Community acquired AKI, n (%)	1,861 (49.7%)	583 (45.2%)	0.76 (0.66-0.87)	0.74 (0.61-0.89)
Cause of AKI^a^				
Ischemic or toxic ATN, n (%)	3,325 (88.8%)	1,140 (88.4%)	0.94 (0.76-1.16)	
Drug toxicity, n (%)	441 (11.8%)	198 (15.4%)	1.65 (1.35-2.02)	
Glomerular disorders, n (%)	464 (12.4%)	304 (23.6%)	4.42 (3.6-5.43)	
COVID-19 related, n (%)	907 (24.2%)	571 (44.3%)	5.01 (4.27-5.88)	
Other, n (%)	527 (14.1%)	230 (17.8%)	1.57 (1.31-1.9)	
Precipitating factors^a^				
Volume depletion, n (%)	2,513 (67.1%)	746 (57.9%)	0.53 (0.46-0.62)	
RAASi, n (%)	318 (8.5%)	106 (8.2%)	0.95 (0.74-1.21)	
Drug toxicity, n (%)	729 (19.5%)	326 (25.3%)	1.72 (1.46-2.03)	
Infection/sepsis, n (%)	2,371 (63.3%)	1,048 (81.3%)	3.72 (3.16-4.37)	
Other, n (%)	197 (5.3%)	60 (4.7%)	0.82 (0.6-1.12)	
Use of IV fluids, n (%)	3,009 (80.4%)	1,056 (81.9%)	1.16 (0.98-1.38)	1.58 (1.2-2.0)
Use of diuretics, n (%)	1,696 (45.3%)	816 (63.3%)	3.08 (2.68-3.55)	1.41 (1.15-1.72)
Use of IV vasopressors, n (%)	1,585 (42.3%)	1,035 (80.3%)	14.11 (11.94-16.67)	3.96 (3.18-4.92)
Use of steroids, n (%)	1,038 (27.7%)	629 (48.8%)	4.76 (4.09-5.55)	1.93 (1.56-2.37)
Use of antibiotics, n (%)	2,930 (78.3%)	1,200 (93.1%)	5.65 (4.47-7.12)	
Need for surgical intervention, n (%)	536 (14.3%)	142 (11.0%)	1.54 (1.25-1.89)	
Use of mechanical ventilation, n (%)	1,516 (40.5%)	1,013 (78.6%)	14.24 (12.07-16.8)	3.29 (2.64-4.1)
Need for dialysis, n (%)	1,255 (33.5%)	784 (60.8%)	6.53 (5.62-7.6)	2.36 (1.93-2.88)

Abbreviations: AKI, acute kidney injury; ATN, acute tubular necrosis; eGFR, estimated glomerular filtration rate; DM, diabetes mellitus; HTN, hypertension; Hgb, hemoglobin; RAASi, renin angiotensin aldosterone inhibitors (angiotensin converting enzyme inhibitor [ACEi] and angiotensin receptor blocker [ARB]); IV, intravenous.

a More than one answer per patient was possible, which explains why the total may be >100%.

b Variables included in the multivariable model: sex; nationality; source of AKI; comorbid conditions (age, eGFR, diabetes, hypertension), baseline Hgb, use of intravenous fluids, diuretics, vasopressors, and steroids, need for ventilation, surgical intervention, and need for dialysis.

**Table 6:** Risk Factors for Higher Need for Dialysis

Variable	Total cohort N = 3,744	Dialyzed N = 1,255	Unadjusted^b^ OR (95% CI)	Adjusted^b^ OR (95% CI)
Male, n (%)	2,218 (59.2%)	760 (60.6%)	1.08 (0.94-1.24)	1.17 (0.98-1.39)
Comorbidities conditions^a^				
Age > 65 y, n (%)	1,827 (48.8%)	544 (43.4%)	0.72 (0.63-0.82)	0.6 (0.5-0.72)
eGFR < 60 mL/min, n (%)	1,563 (41.8%)	481 (38.3%)	0.83 (0.72-0.95)	1.55 (1.28-1.87)
DM, n (%)	2,611 (69.7%)	821 (65.4%)	0.73 (0.63-0.85)	0.86 (0.69-1.06)
HTN, n (%)	2,720 (72.7%)	854 (68.1%)	0.71 (0.61-0.82)	0.82 (0.66-1.02)
Nationality Kuwaiti, n (%)	2,159 (57.7)	693 (55.2)	0.86 (0.75-0.98)	
Baseline Hgb, g/L, mean ± SD	105.1 ± 23.5	103.0 ± 24.9	0.994 (0.991-0.997)	
Community acquired AKI, n (%)	1,861 (49.7%)	675 (53.8%)	1.27 (1.11-1.46)	1.58 (1.33-1.89)
Cause of AKI^a^				
Ischemic or toxic ATN, n (%)	3,325 (88.8%)	1,072 (85.4%)	0.61 (0.5-0.75)	
Drug toxicity, n (%)	441 (11.8%)	196 (15.6%)	1.69 (1.38-2.07)	
Glomerular disorders, n (%)	464 (12.4%)	301 (24.0%)	4.5 (3.66-5.52)	
COVID-19 related, n (%)	907 (24.2%)	531 (42.3%)	4.12 (3.52-4.82)	
Other, n (%)	527 (14.1%)	270 (21.5%)	2.38 (1.97-2.86)	
Precipitating Factors∗				
Volume depletion, n (%)	2,513 (67.1%)	743 (59.2%)	0.58 (0.51-0.67)	
RAASi, n (%)	318 (8.5%)	108 (8.6%)	1.02 (0.8-1.3)	
Drug toxicity, n (%)	729 (19.5%)	299 (23.8%)	1.49 (1.26-1.76)	
Infection/sepsis, n (%)	2,371 (63.3%)	936 (74.6%)	2.15 (1.85-2.5)	
Other, n (%)	197 (5.3%)	107 (8.5%)	2.48 (1.86-3.31)	
Use of IV fluids, n (%)	3,009 (80.4%)	972 (77.5%)	0.76 (0.64-0.9)	
Use of diuretics, n (%)	1,696 (45.3%)	850 (67.7%)	4.07 (3.52-4.7)	2.52 (2.12-3.0)
Use of IV vasopressors, n (%)	1,585 (42.3%)	917 (73.1%)	7.39 (6.34-8.61)	2.56 (2.05-3.18)
Use of steroids, n (%)	1,038 (27.7%)	617 (49.2%)	4.75 (4.07-5.53)	1.82 (1.5-2.2)
Use of antibiotics, n (%)	2,930 (78.3%)	1,131 (90.1%)	3.49 (2.84-4.29)	
Need for surgical intervention, n (%)	536 (14.3%)	138 (11.0%)	1.54 (1.25-1.89)	
Use of mechanical ventilation, n (%)	1,516 (40.5%)	929 (74.0%)	9.23 (7.89-10.79)	4.02 (3.23-5.0)

Abbreviations: AKI, acute kidney injury; ATN, acute tubular necrosis; eGFR, estimated glomerular filtration rate; DM, diabetes mellitus; HTN, hypertension; Hgb, hemoglobin; RAASi, renin angiotensin aldosterone inhibitors (angiotensin converting enzyme inhibitor [ACEi] and angiotensin receptor blocker [ARB]); IV, intravenous.

a More than one answer per patient was possible, which explains why the total may be >100%.

b Variables included in the multivariable model: sex; nationality; source of AKI; comorbid conditions (age, eGFR, diabetes, hypertension), baseline Hgb, use of intravenous fluids, diuretics, vasopressors, and steroids, need for ventilation, and surgical intervention.

**Table 7:** Risk Factors Associated With Higher Rates of Lack of Recovery of Kidney Function in Patients who Were Alive at 30 Days (*N* = 2,455)

Variable	Alive N = 2,455	No recovery N = 426	Unadjusted^b^ OR (95% CI)	Adjusted^b^ OR (95% CI)
Male, n (%)	1,465 (59.7%)	255 (17.4%)	0.99 (0.8-1.23)	1.18 (0.92-1.51)
Comorbid conditions∗				
Age > 65 y, n (%)	1,151 (46.9%)	200 (17.4%)	0.99 (0.8-1.22)	0.91 (0.7-1.19)
eGFR < 60 mL/min, n (%)	1,143 (46.6%)	235 (21.2%)	1.5 (1.21-1.85)	1.26 (0.97-1.64)
DM, n (%)	1,734 (70.6%)	307 (17.7%)	1.06 (0.84-1.34)	0.96 (0.7-1.3)
HTN, n (%)	1,815 (73.9%)	337 (18.6%)	1.37 (1.06-1.76)	1.51 (1.08-2.12)
Nationality Kuwaiti, n (%)	1,400 (57.0)	261 (18.6)	1.21 (0.98-1.5)	
Baseline Hgb level, g/L mean ± SD	106.5 ± 23.9	98.8 ± 20.1	0.98 (0.975-0.986)	0.984 (0.978-0.989)
Community acquired AKI, n (%)	1,278 (52.1%)	255 (20.0%)	1.44 (1.17-1.78)	1.3 (1.02-1.66)
Cause of AKI^a^				
Ischemic or toxic ATN, n (%)	2,185 (89.0%)	384 (17.6%)	1.09 (0.77-1.54)	
Drug toxicity, n (%)	243 (9.9%)	52 (21.4%)	1.32 (0.95-1.84)	
Glomerular disorders, n (%)	160 (6.5%)	61 (38.1%)	3.23 (2.3-4.53)	
COVID-19 related, n (%)	336 (13.7%)	102 (30.4%)	2.39 (1.84-3.11)	
Other, n (%)	297 (12.1%)	56 (18.9%)	1.11 (0.81-1.52)	
Precipitating factors^a^				
Volume depletion, n (%)	1,767 (72.0%)	287 (16.2%)	0.75 (0.6-0.94)	
RAASi, n (%)	212 (8.6%)	28 (13.2%)	0.7 (0.46-1.05)	
Drug toxicity, n (%)	403 (16.4%)	77 (19.1%)	1.14 (0.87-1.5)	
Infection/sepsis, n (%)	1,323 (53.9%)	273 (20.6%)	1.63 (1.32-2.03)	
Other, n (%)	137 (5.6%)	25 (18.3%)	1.06 (0.67-1.65)	
Use of IV fluids, n (%)	1,953 (79.6%)	315 (16.1%)	0.66 (0.51-0.84)	
Use of diuretics, n (%)	880 (35.9%)	224 (25.5%)	2.29 (1.85-2.83)	
Use of IV vasopressors, n (%)	550 (22.4%)	151 (27.5%)	2.22 (1.85-2.83)	
Use of steroids, n (%)	409 (16.7%)	129 (31.5%)	2.69 (2.11-3.42)	1.71 (1.28-2.29)
Use of antibiotics, n (%)	1,730 (70.5%)	334 (19.3%)	1.6 (1.25-2.05)	
Need for surgical intervention, n (%)	394 (16.1%)	41 (10.4%)	1.99 (1.41-2.8)	1.7 (1.16-2.48)
Use of mechanical ventilation, n (%)	503 (20.5%)	145 (28.8%)	2.38 (1.89-3.0)	
Need for dialysis, n (%)	471 (19.2%)	235 (49.9%)	9.24 (7.31-11.67)	8.0 (6.3-10.3)

Abbreviations: AKI, acute kidney injury; ATN, acute tubular necrosis, eGFR, estimated glomerular filtration rate; DM, diabetes mellitus; HTN, hypertension; Hgb, hemoglobin; RAASi, renin angiotensin aldosterone inhibitors (angiotensin converting enzyme inhibitor [ACEi] and angiotensin receptor blocker [ARB]); IV, intravenous.

a More than one answer per patient was possible, which explains why the total may be >100%.

b Variables included in the multivariable model: sex; nationality; source of AKI; comorbid conditions (age, eGFR, diabetes, hypertension), baseline Hgb, use of intravenous fluids, diuretics, vasopressors, and steroids, need for of ventilation, surgical intervention, and need for dialysis.

## Discussion

AKI can result from an acute illness that a patient presents to a hospital with or can be iatrogenic (eg, because of nephrotoxins, surgery, etc.). In this article, a large cohort of AKI patients from the Middle East, an underreported region of the world, was recruited. We report an incidence of 3.2% of AKI in all hospitalized patients in participating hospitals over a 1-year period. AKI incidence was higher in critically ill patients, accounting for 19.5% of ICU admissions. The incidence of AKI according to different studies is between 3% and 18%.^9^ Kuwait has a large ethnically diverse expatriate community, representing 66% of the total population. AKI was reported more frequently in Kuwaitis than non-Kuwaitis (Table 4) despite the fact the Kuwaitis represent only one-third of the population and despite the fact that non-Kuwaitis have a lower income and a restricted access to public health care services as detailed in the Introduction section. This is probably because Kuwaitis were older with more comorbid conditions and lower baseline eGFR, as shown in Table 1. About 89% of AKI cases were attributed to acute tubular necrosis, and 24.2% were associated with COVID-19 infection, with no difference between the 2 groups. The study Program to Improve Care in Acute Renal Disease (PICARD) looked at the etiology of AKI in over 600 critically ill patients in 5 ICUs in the United States and found out that 75% of the cases were attributed to acute tubular necrosis (with two-thirds ischemic and one-third nephrotoxic in nature).^10^ With regards to COVID-19-associated AKI, reported rates vary depending on population studied, methodology, and timing of reporting during the pandemic. Our study was conducted in 2021, with high rates of COVID-19 infections in the first half of that year and a low rate of infections in the second half of the year. The prevalence of COVID-19-associated AKI in a meta-analysis of about 13,000 patients was 17% with a wide range in the included studies of 0.5% to 80%.^11^ Volume depletion and/or sepsis were the most common precipitating factors for AKI. More cases of volume depletion occurred in the non-Kuwaiti group, whereas sepsis was more prevalent in Kuwaitis. The risk for AKI was particularly high in patients of advanced age (>65 years) and with common comorbid conditions, such as DM, HTN, and cardiovascular disease. These risks are universal and have been reported in different populations in various studies worldwide.^12^, ^13^, ^14^, ^15^, ^16^ All these comorbid conditions were significantly more prevalent in Kuwaiti patients, especially those age > 65 years, with levels of prevalence almost twice as much as that noted in non-Kuwaitis.

Management of AKI is mainly supportive and directed toward the cause. In some cases, kidney replacement therapy is warranted. There was no difference between the 2 groups in either the proportion of patients in the ICU or those requiring mechanical ventilation. However, Kuwaitis represent 60.4% of the ICU patients with AKI as shown in Table 4; this is because Kuwaitis were older with higher comorbid condition burden (DM, HTN, and cardiovascular disease), resulting in a more severe acute illness and, thus, a higher need for ICU. As stated in the Introduction section, hospital admission for non-Kuwaitis is unrestricted and that includes ICU admission. Vasopressors and inotropic support were administered in 42.3% of all patients, slightly more often in the Kuwaiti group. Dialysis was required for 33.5% of AKI patients, mainly for hyperkalemia and acid–base imbalance; this proportion did not differ between the groups. However, volume overload refractory to medical therapy was significantly more frequent indication for dialysis in Kuwaitis than non-Kuwaitis.

Total mortality rate was high at 34.4%. A retrospective population-based study found a mortality rate of 35% with AKI.^17^ The AKI-related mortality rate in adults in a meta-analysis of 110 studies conducted worldwide was 24%. However, the mortality rates for AKI stage 2, AKI stage 3 and AKI requiring dialysis in that meta-analysis were 28.5%, 47.8%, and 49.4%, respectively.^9^ Another single center study conducted over 1 year in the United Kingdom reported a high AKI-associated mortality rate of 22%, despite the fact that 62% of patients had AKI stage 1, and only 2.5% needed dialysis (ie, mild disease).^18^ The majority of our mortality comes from the ICU and from the dialysis pools. AKI-related mortality outside the ICU in our cohort was only 17.3%. The mortality of adult non-ICU hospitalized patients reported in the literature is 10%-20%.^19^ The pooled mortality rate of COVID-19 infected patients with AKI from 9 studies was 52% (range, 7%-100%).^11^ COVID-19 infection was not a significant risk factor for mortality in our cohort. There is an inverse relationship between AKI-associated mortality and a country’s expenditure on health care, with higher mortality in lower income countries and vice versa.^20^ AKI in low-income countries carries a relatively higher mortality rate of 27% at 6 months, which is likely because of delays in initial diagnosis and management; late presentations with subsequent higher severity of the illness and higher likelihood of requiring dialysis and critical care; and in some cases, an inability to deliver dialysis treatment to those who need it.^7^^,^^20^^,^^21^ A systematic review of 17 studies in sub-Saharan Africa reported a rate of 32% of AKI-associated mortality (with mean study duration of 5 years).^21^ Kuwait is considered to be a high-income country,^22^ and our incidences are comparable to those of similar high-income countries. However, several characteristics of AKI in the non-Kuwaiti group were similar to those reported in low-income countries, including more community acquired AKI, with volume depletion as the most common cause (with less need for diuretics and less volume overload as an indication for kidney replacement therapy), and a higher prevalence in males (> 60% of expatriates in Kuwait are male).^3^^,^^23^ The low income of non-Kuwaitis and the nature of their jobs, which may entail long hours, physically demanding work, and lack of shelter from the sun in the hot and dry weather during the long summer, and less access to air-conditioning at home or at work, are probably responsible for this difference. However, despite the restricted access of non-Kuwaitis to the public health care system, there were no significant differences between the 2 groups in mortality and kidney recovery rates at 30 days. The younger age, the higher mean baseline eGFR, the lower comorbid condition burden, and the higher rate of community acquired AKI in non-Kuwaitis could have compensated for the disadvantaged status and resulted in similar outcomes to Kuwaitis because community acquired AKI is associated with lower mortality.^24^ As discussed in the Introduction, expatriates in Kuwait need to pay fees when visiting MoH facilities, whether primary care clinics or hospitals, including emergency department visits. However, when hospital admission is required, they receive unrestricted medical care.^4^ This unrestricted admission policy may also explain the comparable outcomes of non-Kuwaitis despite their disadvantages to those of Kuwaitis. Community acquired AKI was associated with lower risk of mortality in our cohort but with higher need for dialysis and with less rates of kidney recovery. A meta-analysis of 15 studies including more than 46,000 adult patients reported lower mortality with community acquired AKI in comparison to hospital-acquired AKI as in our study, but similar rates for need for dialysis and kidney recovery.^24^ We reported significantly higher mortality with advanced age, the use of vasopressors, the need for mechanical ventilation, and the need for dialysis, which are similar findings reported in the literature.^25^, ^26^, ^27^, ^28^, ^29^ AKI patients who required vasopressors and mechanical ventilation and those of advanced age have a significantly higher likelihood of needing dialysis. The higher likelihood of need for dialysis and/or mortality in AKI patients on inotropic support and/or mechanical ventilation may be indicative of the severity of illness. The PICARD study showed that AKI can be associated with multiorgan failure even in patients who did not need dialysis, and that the majority of patients who required dialysis were critically ill patients admitted to ICU.^10^ Furthermore, our AKI patients who required dialysis and mechanical ventilation had a significantly higher likelihood of lack of kidney function recovery. These are all well known risk factors studied previously and have been reported in the literature.^23^^,^^30^^,^^31^ Sepsis, as a precipitating factor for AKI and as a cause of death, was more common in Kuwaitis than in non-Kuwaitis. However, sepsis was not associated with a worse outcome. Rates of recovery of kidney function at 30 days, whether complete, partial or none, were similar in the 2 groups. The lower mortality rate in patients with eGFR <60 mL/min was reported in other studies. A study from Germany reported lower in-hospital mortality following AKI in patients with pre-existing advanced CKD compared with patients without pre-existing CKD (13.4% vs 21.7%).^32^ Similarly in the PICARD study, AKI superimposed on CKD resulted in lower mortality rates than new-onset AKI (31% vs 41%).^10^ We hypothesize that patients with eGFR < 60 mL/min are followed more frequently and advised to seek medical attention early if unwell. This facilitates earlier intervention, earlier discontinuation, and/or avoidance of injurious agents, all resulting in favorable outcomes. In addition, CKD patients could have adapted to the impaired kidney function and are less likely to be severely affected by hyperkalemia compared with patients with normal kidney function. Other possible factors may include higher rates of COVID-19 infection in patients with eGFR > 60 mL/min. We did not collect data on AKI following coronary angiography and cardiac surgery. Our cohort had a low mean hemoglobin level that was comparable between both groups and was only slightly associated with lack of recovery but not with mortality or dialysis need. Anemia is common in surgical patients for several reasons, and published reports have linked anemia, whether pre- or postoperatively, with AKI and increased 30-day mortality.^33^, ^34^, ^35^ However, we found no studies evaluating anemia as a risk factor for AKI in the nonsurgical population.

Our study is limited because of its observational nature, potential selection bias, and short follow-up of 30 days. However, it is one of the largest prospective studies from the Middle East on AKI that evaluated the effect of socioeconomic status and access to health care on AKI outcomes.

In conclusion, this was a large multicenter, multinational study from the Middle East showing that AKI is associated with a high need for dialysis, high 30-day mortality rates, and low rates of complete kidney recovery, similar to what has been reported in the literature. It also showed lack of association between lower eGFR and poorer outcomes in AKI, and this finding has been reported before in different populations. Non-Kuwaitis represent 66% of the population, have a lower socioeconomic status, and a restricted access to MoH laboratory, radiology, and pharmacy services compared with Kuwaitis; however, this population accounted for <50% of hospitalized AKI patients and had similar mortality rates and complete kidney recovery rates as Kuwaitis, despite a higher need for dialysis, probably because they were younger, had higher baseline eGFR, and fewer comorbid conditions, but not because of admission policy. However, non-Kuwaitis had higher rates of community acquired AKI and AKI in the hot summer months and more cases of volume depletion, probably related to occupation and access to air-conditioning.
